# Effects of Extreme Temperatures on Mortality and Hospitalization in Ho Chi Minh City, Vietnam

**DOI:** 10.3390/ijerph16030432

**Published:** 2019-02-02

**Authors:** Tran Ngoc Dang, Yasushi Honda, Dung Van Do, Anh Lan Thi Pham, Cordia Chu, Cunrui Huang, Dung Phung

**Affiliations:** 1 Institute of Research and Development, Duy Tan University, Da Nang 550000, Vietnam; 2 Faculty of Public Health, University of Medicine and Pharmacy in Ho Chi Minh City, Ho Chi Minh 70000, Vietnam; dvdung@ump.edu.vn (D.V.D.); lanh2804@gmail.com (A.L.T.P.); 3 Faculty of Health and Sport Sciences, University of Tsukuba, Tsukuba 305-8577, Japan; honda.yasushi.fn@u.tsukuba.ac.jp; 4 Centre for Environment and Population Health, Griffith University, Brisbane 4001, Australia; c.chu@griffith.edu.au; 5 School of Public Health, Sun Yat-sen University, Guangzhou 510000, China; huangcr@mail.sysu.edu.cn; 6 School of Medicine, Nathan Gold Coast Campus, Griffith University, Nathan QLD 4111, Australia

**Keywords:** temperature-related mortality, temperature-related hospitalization, main effect, added effect, heatwaves, Vietnam

## Abstract

There is a lack of research focusing on the association of temperature with mortality and hospitalization in developing countries with tropical climates and a low capacity to cope with the influences of extreme weather events. This study aimed to examine and compare the effect of temperature, including heat waves, on mortality and hospitalization in the most populous city of Vietnam. We used quasi-Poisson time series regression coupled with the distributed lag non-linear model (DLNM) to examine the overall pattern and compare the temperature-health outcome relationship. The main and added effects of heat waves were evaluated. The main effect of heat waves significantly increased the risk of all cause-specific mortality. Significant main effects of heat waves on hospitalization were observed only for elderly people and people with respiratory diseases (elderly, relative risk (RR) = 1.28, 95% confidence interval (CI) = 1.14–3.45; respiratory diseases, RR = 1.3, 95% CI = 1.19–1.42). The RRs of the main effect were substantially higher than those of the added effect in mortality; the same was applicable for hospitalizations of people with respiratory diseases and elderly people. The findings of this study have important implications for public health adaptation and prevention program implementation in the protection of residents from the adverse health effects of temperature.

## 1. Introduction

Global warming, a phenomenon of climate change, the rate of which has been accelerated by increasing anthropogenic greenhouse gas emissions, is contributing to a higher frequency, intensity, and duration of extreme weather events, such as heat waves [1]. High temperatures are significantly associated with both morbidity [2,3,4] and mortality [5,6,7,8], and the associated adverse health outcomes encompass a wide range of communicable [9,10,11,12,13] and non-communicable diseases [14,15,16,17]. The impact of high temperatures is substantially amplified in megacities with large populations and building densities due to the thermal properties of the built environment (the so-called urban heat island phenomenon).

Studies in temperature–health effects are still open to fulfilling a couple of knowledge gaps. First, while there is sufficient evidence on the effect of high temperatures on mortality, worldwide, the evidence on the effect of high temperatures on hospitalization is less conclusive [18]. For instance, studies have demonstrated that high temperatures are associated with increases in the rates of hospital admission due to cardiorespiratory diseases in several cities in the United States of America (USA) [15,19,20]. However, the relationship between high temperatures and cardiovascular disease (CVD) admissions was not significant in some European cities [3,21,22] and in some cities in the USA [23]. Second, the contrasting pattern of CVD hospitalizations and mortality in relation to heat waves raised a hypothesis that deaths due to CVD occur rapidly among people before they reach a hospital [24]. However, most of the above-mentioned studies used different analytic methods and populations; therefore, it is difficult to compare the results. It is important to conduct further studies to examine the hypothesis using data on mortality and hospitalization from the same population and applying the same statistical analyses for comparison. The contrasting patterns of CVD hospitalization and mortality in relation to heat also open the question “Do the patterns of different types of diseases (e.g., cardiovascular, respiratory) and different population characteristics (e.g., men, women, elderly people, etc.) vary in response to different types of heat (e.g., high temperature and heat waves)?”. Finally, most previous studies in this context were implemented in developed countries with a temperate climate. There is a lack of research on the relationship of temperature with mortality and hospitalization in developing countries that have a tropical climate and a low capacity to cope with the influences of extreme weather events [4,21,25,26], even though a non-uniform spatial pattern in heat-related health risk has been strongly theoretically supported based on a previous study [2].

This study aims to examine the main and added effects of heat waves on mortality and hospitalization. In addition, we also compare the relationship between heat-mortality and heat-hospitalization.

## 2. Materials and Methods

### 2.1. Study Area

Ho Chi Minh City (HCMC) is located in the south of Vietnam and has a “tropical wet and dry” climate (Köppen-Geiger classification). HCMC experiences a high annual average temperature and two distinct seasons: a rainy season and a dry season. The rainy season usually lasts from May to November, with an average rainfall of about 1800 mm during the season, and about 150 rainy days per year [27]. The dry season extends from December to April, and the period with the highest temperature is from March to May (i.e., sometimes March–May is considered ‘summer’ in HCMC). The annual average temperature is 28 °C, with few variations throughout the year, and the city experiences between 2400 and 2700 h of sunshine per year [27]. HCMC is one of the most populous cities in Vietnam, with a total population of more than 7 million, accounting for 8.4% of the total population of Vietnam; the population density is approximately 2660 people per km^2^ [28].

### 2.2. Data Source and Quality

Daily mortality data from 322 community health centers in 24 districts of HCMC were collected from the national system (the A6 mortality reporting system) from 1 January 2010 to 31 December 2013. The A6 mortality system has been described elsewhere [29]; data from this system have been validated in previous studies and showed good completeness and accuracy, particularly in regard to circulatory disease, cancer, and injury cases [30,31]. The final mortality data in this study included 101,959 decedents, with information on the date of death, sex, age, and cause of death as classified by the 10th Revision of the International Classification of Disease (ICD10) code. In this study, cause-specific mortality included CVDs (i.e., ICD10 code I00-99) and respiratory diseases (i.e., ICD10 code J00-99).

Data on hospital admissions included daily counts for non-external causes, CVDs (I00-99; excluding acute rheumatic fever, I00-02, and chronic rheumatic heart diseases, I05-09), and respiratory diseases (J00-99; excluding lung diseases due to external agents, J60-70). We obtained admission data for the period from 1 January 2010 to 31 October 2013 from the hospital records of the two largest hospitals in HCMC: Gia Dinh People’s Hospital and 115 People’s Hospital. These multi-faculty hospitals have 1200 and 1600 beds, respectively. Data extracted from the admission records included those on primary and discharge diagnoses (ICD-10 codes), date of admission, date of discharge, age, sex, and the district of residence of individual patients. To avoid exposure misclassification, patients from locations other than HCMC were excluded.

Daily weather data were obtained from the National Oceanic and Atmospheric Administration’s National Climate Data Center for the same period with mortality and hospitalization data. The data comprise those on daily minimum, average, and maximum temperatures (°C), and relative humidity (%) collected at the Tan Son Nhat airport weather monitoring station [32].

This study was approved by the Griffith University Human Research Ethics Committee (GU Ref No: ENV/23/15/HREC) and a support letter from the Health Environment Management Agency, Vietnam Ministry of Health (No: 937/MT-SKCĐ, 2013).

### 2.3. Definition of a Heat Wave

While there is no global consensus on the definition of a ‘heat wave’, it is commonly defined as a few consecutive days with high temperatures above a certain threshold [33]. In this study, heat waves were defined using a combination of intensity (≥97th percentile of the daily average temperature, i.e., 30.9 °C) and duration (≥2 consecutive days). This definition is consistent with that used in some previous studies [34,35].

Several recent studies have described the heat wave effect as a sum of two contributions: the intensity effect due to the independent effects of daily hot temperatures (i.e., main effect), and the duration effect due to sustained heat wave days (i.e., added effect) [34,36,37]. Therefore, in this study, we examined both the main and added effects of heat waves on mortality and hospitalization using a time series regression model, as shown in detail below.

### 2.4. Statistical Model

We used a quasi-Poisson time series regression model linking daily mortality or daily hospitalization (i.e., outcomes or responses) with daily average temperature (i.e., exposure) [38]. To control for long-term trends and seasonality, we used a natural cubic spline function of time with 7 degrees of freedom (df) per year. In addition, to account for the potential non-linear relation between temperatures and health outcomes, we applied a distributed lag non-linear model (DLNM) using a cross-basis function of multiple lag-day temperatures [39,40]. The parameters of this cross-basis function followed the specifications indicated in a previous study [41], which included a quadratic B-spline with two internal knots placed at equally spaced values of the temperature in the exposure-response dimension, and a natural cubic B-spline with an intercept and three internal knots placed at equally spaced values of the log scale of lags in a lag-response dimension. The allowed maximum lags in this study were up to 7 days, considering the fact that the hot temperature effects were acute and possibly affected by mortality displacement [5]. To examine the independent effects of heat waves (i.e., main and added effects) on outcomes, we introduced into the model at the same time the continuous average temperature variable and the heat wave indicator variable that has two values: ‘1’ if a heat wave occurred and ‘0’ if otherwise. The general model is:

Yt~quasi-Poisson (µt) Log (Yt) = α + β1 * Tt,l + β2 * HW + β3 * DOW + β4 * NCS (Time, 7 df/year)(1)

where Yt denotes the daily count of outcomes (mortality or hospitalization) on day t; l signifies the lag days; Tt,l signifies a matrix obtained by applying the “cross-basis” DLNM functions to the mean temperature; HW is a heat wave indicator (i.e., ‘1’ if a heat wave, otherwise ‘0’); DOW signifies the day of the week; NCS is a natural cubic spline function; and Time is a variable that takes consecutive numbers from ‘1’ on the first day of observation to ‘1461’ on the final day within the observation period (2010‒2013).

The main effect of heat waves was then calculated as the relative risk (RR) of the median value of temperature distribution among heat wave days (HW = 1) compared to the minimum mortality/or hospitalization temperature (MMT) (i.e., the temperature at which the risk is lowest). The added effect of heat waves was calculated as the RR between heat wave days and non-heat wave days (i.e., exp(β2) in Equation (1)).

We also performed sensitivity analyses to test these modeling choices, in which we simplified the lag structure by fitting the moving average of temperature series over lag 0–1,0–3, and 0–7 days and changing the number of knots for temperature and lag days; and performed sensitivity analyses with different heat wave intensity definitions (i.e., 97th, 98th, and 99th percentile) and duration (i.e., 2 days, 4 days). All analyses were performed using R software 3.2.2., the “dlnm” package [40]. The R code to reproduce the results of this study can be obtained by contacting the first author.

## 3. Results

### 3.1. Descriptive Statistics

The total mortality count from 2010 to 2013 in HCMC was 101,959, including 22,218 cases (21.8%) attributed to CVDs, and 8804 (8.63%) to respiratory diseases. A large proportion of the deceased people were older than 65 years, accounting for 58.2% of all deaths. The mortality values in the male and female cases were 54.6% and 45.4% respectively. A total of 310,045 all-cause hospitalizations were recorded during the study period. The highest proportion (70%) of hospitalizations were attributed to elderly people, while a higher proportion of female patients than male patients were hospitalized (51.8% vs. 48.2%). On average, the all-cause daily mortality and daily hospitalization values were 70 and 221 cases, respectively. Table 1 shows the descriptive statistics of daily weather condition, daily mortality, and daily hospitalization.

The weather in HCMC was hot year-round, with a mean daily average temperature of 28.4 °C, ranging from 23 to 32 °C. Table 2 displays data on the intensity and duration of the heat waves in HCMC during the study period. The longest heat wave lasted 16 days from 5–21 May 2010, in which the median temperature was 31.75 °C, ranging from 30.95 to 32.1 °C.

**Figure 1:**
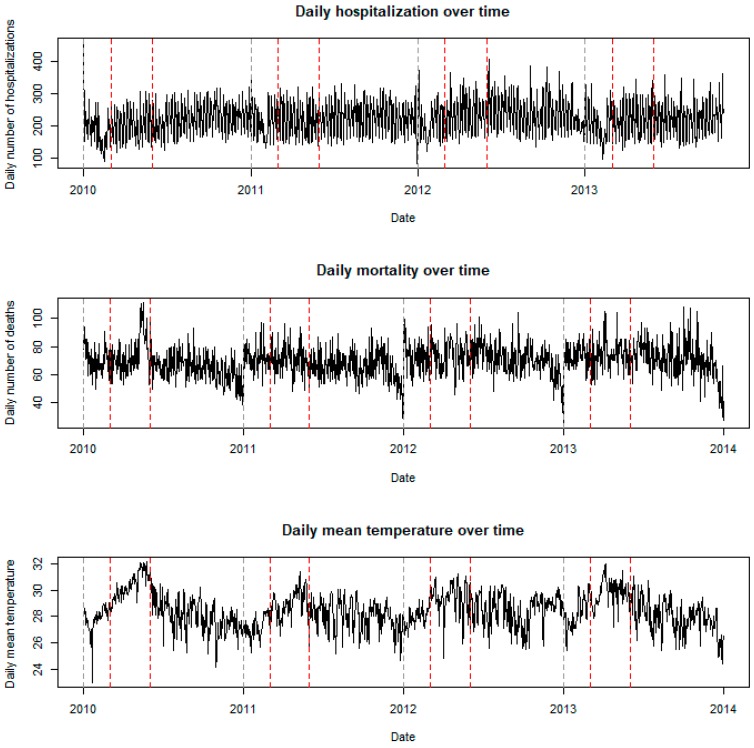
Time series plot of the daily basis of all-cause mortality, all-cause hospitalization, and temperature in Ho Chi Minh City from 2010 to 2013. The red lines indicate the summer period from March to May.

Figure 1 shows the time series plot of the daily basis of all-cause mortality, all-cause hospitalization, and temperature. In the summer months in 2010 and 2013, the temperatures were very high (i.e., on average above 31 °C, and sometimes reached 32 °C), indicating that heat waves occurred during these periods. The observed daily mortality counts were also high during the summer months in 2010 and 2013; this implies the heat wave occurrence increased the mortality risk. The effect of heat waves on hospitalization, however, was not obvious.

### 3.2. The Short-Term Relationship between Temperature and Health Outcomes

**Figure 2:**
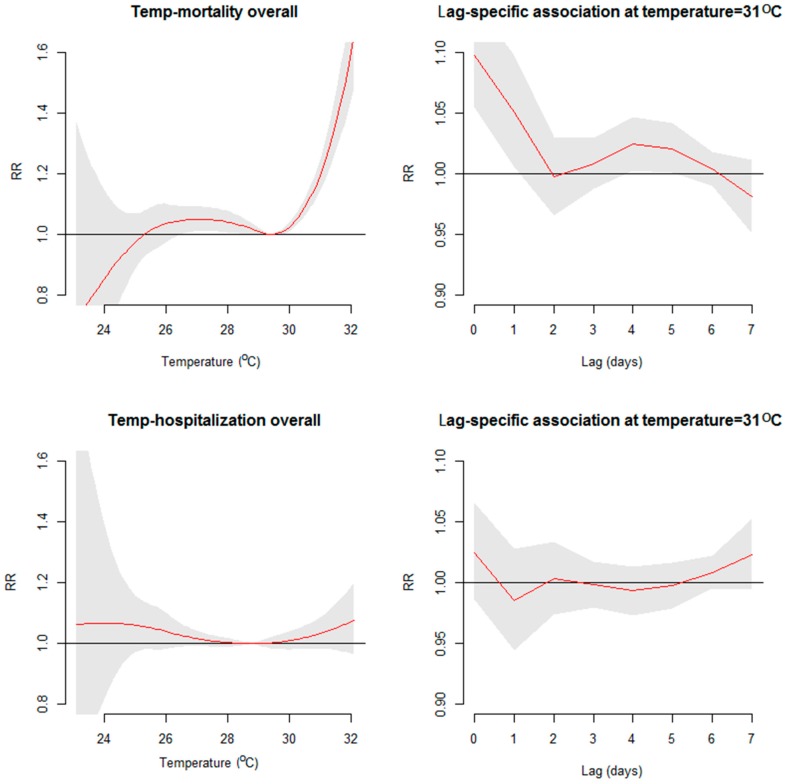
Short-term association between all-cause mortality, all-cause hospitalization, and temperature.The red curves indicate cumulative relative risk (RR), and the grey areas indicate the 95% confidence intervals.

The overall short-term associations between mortality, hospitalization, and temperature are shown in Figure 2. The temperature–mortality relationship appeared J-shaped with an MMT of 29.4 °C (i.e., temperatures above 29.4 °C increased the risk of mortality), whereas the temperature–hospitalization relationship showed a modest increase in the risk of hospitalization at very high temperatures (the increase was not statistically significant). The hot effect was acute for mortality (the effect of 31 °C occurred on day 0 and lasted two days), whereas the hot effect was neither acute nor significant for hospitalization.

We performed sensitivity analyses to check the robustness of the model. The alternative models for the lag structure are shown in Figure S1 for mortality and Figure S2 for hospitalization, and alternative models for a number of knots for temperature and lag days are shown in Figure S3. The overall temperature–mortality and temperature–hospitalization curves of the alternative models were relatively similar (especially in terms of the heat-related association), implying that our results are robust and unlikely to be affected by the modeling choices.

### 3.3. Comparability of the Temperature Effects on Mortality and Hospitalization

**Figure 3:**
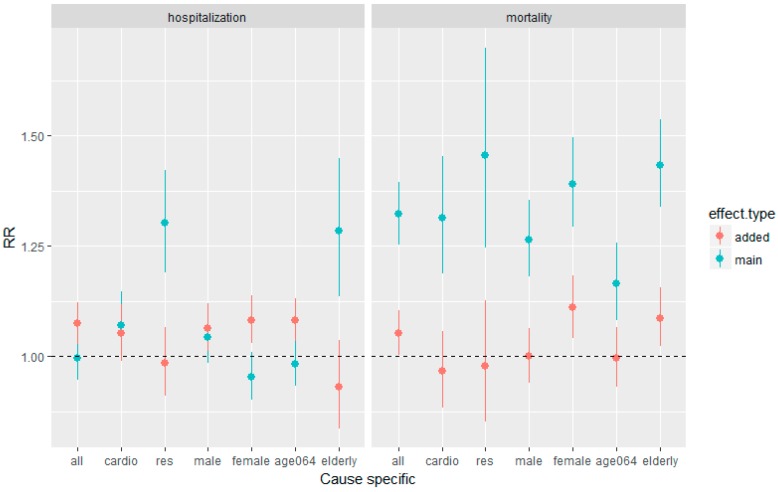
Main and added effect of heat waves on hospitalization and mortality cause specific. The left panel is for hospitalization and the right panel is for mortality. The blue point are the RRs of main effects with 95% confidence intervals and the red point are the RRs of added effects with 95% confidence intervals.

The main and added effects of heat waves are presented in Figure 3. It was observed that the main effect of heat waves significantly increased the risk of mortality in all categories, in which the RRs were the highest for the mortality of people with respiratory diseases and elderly people (respiratory mortality, RR = 1.45, 95% confidence interval (CI) = 1.25–1.70; elderly people mortality, RR = 1.43, 95% CI = 1.34–1.53). The main effect of heat waves on hospitalization, however, was only significant for the hospitalizations of people with respiratory diseases and elderly people (respiratory hospitalization, RR = 1.3, 95% CI = 1.19–1.42; elderly people hospitalization, RR = 1.28, 95% CI = 1.14–3.45). The RRs of the main effect was substantially higher than those of the added effect in mortality; the same was applicable to hospitalizations of people with respiratory diseases and elderly people. We observed elevated risks of the added effect in hospitalization in both the male and female groups (male hospitalization, RR = 1.06, 95% CI = 1.01–1.12; female hospitalization, RR = 1.08, 95% CI = 1.03–1.14), but only elevated risks of the added effect in mortality in the female group (female mortality, RR = 1.11, 95% CI = 1.04–1.18). It is worth noting that the added effect was significant in the hospitalization group with younger age, but not significant in the mortality group with younger age (0–64 years at hospitalization, RR = 1.08, 95% CI = 1.03–1.13).

The sensitivity results of the different heat wave definitions are shown in Figure S4 for mortality and Figure S5 for hospitalization. The patterns of the main and added effects of the heat waves were quite consistent across the definitions, indicating that the results are unlikely to be affected by the heat wave definitions.

## 4. Discussion

This is the first comprehensive study to examine the effects of temperatures on health outcomes using both mortality and hospitalization data in Vietnam. The study reports findings on the patterns of the temperature–health risk relationship. Overall, the results of this study revealed that increased temperatures above the threshold of 29 °C were associated with an elevated risk of mortality but not hospitalization. The same applied to the main effect of temperature (i.e., intensity effect) during heat waves, except in the case of elderly people and people with respiratory diseases. In the same geographical area, despite a lack of studies on mortality, a study conducted by Phung et al. (2016) illustrated that 1 °C increases in the average temperature were associated with 1.3% increases in the risk of hospital admission for all causes in the Mekong Delta region [18]; significant effects of temperature were observed in hospital admissions due to infectious and respiratory diseases. However, the relationship between temperature and CVD admission was insignificant in that study, similar to the findings of our study. The discrepancy in the magnitude of risk between hospitalizations and mortality during heat waves has been reported in other regions as well. For example, during the 1995 Chicago heat waves, the all-cause mortality increased by 147%, whereas the hospital admission rate increased by only 11% [42]. In Greater London, the United Kingdom, during the 1995 6-day heat wave, the daily mortality values rose by 10.8% (95% CI 2.8 to 19.3), while the daily hospitalization rate increased by 2.6% (95% CI −2.2 to 7.6) [43]. This discrepancy may be attributed to the fact that many deaths occur rapidly or among isolated people before they reach the hospital during heat waves [24,44]. In the Chicago heat waves, many deaths were observed among people living alone or who had a lack of social contacts [44]. The presence of working home air-conditioning, taking extra showers, using fans, and visiting cool environments are associated with a lower risk of death during heat waves [45]. A study conducted by Nitschke et al. found that the total mortality, as well as disease- and age-specific mortality values, did not increase during heat waves in Adelaide, potentially due to the high prevalence of air conditioning (82%) [46]. In HCMC, the discrepancy between mortality and hospitalization during heat waves indicates that the population is currently not well-adapted to heat waves. There is a need for further studies investigating the specific factors associated with a higher risk of death during heat waves, and to provide potential risk-reducing interventions in the setting of heat waves in HCMC.

This study also revealed that the main effect of heat waves (i.e., intensive effect) was stronger than the added effect in mortality. This result is similar to that observed in a study conducted in 108 communities in the USA, in which the added effect was small and only apparent after 4 consecutive days [34]. Another study in the four communities of Guangdong province, China, found that the main effect was greater than the added effect (i.e., 8.2% vs. 0%) [37]. Our results have implications for the heat-health warning system (HWS) in HCMC. It is very important for the HWS to provide alerts on days with high temperatures when heat waves occur rather than basing alerts on the duration of the heat waves. However, the added effects of heatwave were significant for elderly and female mortality. Therefore, for vulnerable groups (i.e., elderly and female populations) the alerts of HWS based on durations of the heat waves are also important to prevent deaths from heatwaves even if/when the temperatures are not so high during the heatwaves.

The significant positive relationship observed between temperature and the risk of respiratory disease and elderly people-related mortality and hospitalization can be explained by some possible mechanisms, although the causal effect of this relationship is not well understood. First, it is known that chronic obstructive pulmonary disease (COPD) exacerbations are among the most common reasons for respiratory disease admissions among elderly people [47]. These acute episodes are associated with airway and systemic inflammation as well as cardiovascular comorbidity, and may be facilitated by heat exposure. Under extremely hot weather conditions, COPD patients may hyperventilate [48], increasing the possibility of dynamic hyperinflation, leading to dyspnea and mechanical and cardiovascular effects. Moreover, elderly people with COPD may be unable to dissipate excess heat through circulatory adjustment, and exposure to high temperatures may lead to the risk of developing pulmonary vascular resistance secondary to the peripheral pooling of blood or hypovolemia [48]. The underlying mechanism of the temperature–respiratory health relationship should be further studied for a better understanding of the pathophysiology and clinical course of heat-related illness. The findings of this study are in line with this possible mechanism since the highest effects of temperature were observed among elderly people.

This study has some limitations. First, while the mortality data are considered to be representative of the research location, the hospital admission data were obtained from two multi-faculty hospitals, which may not reflect the hospitalizations of the whole city. Moreover, these hospitals provide a higher level of curative treatment, so minor cases that were admitted to private clinics or lower-level hospitals (i.e., district hospitals) may have been missed. Second, this study could not examine the effects of high temperatures on individual cause-specific diseases, focusing instead on groups of diseases (all-cause, respiratory diseases, and CVDs) due to the limited number of cases. A previous study [18] indicated that temperature may have different effects by disease type, especially in the case of CVDs. For example, a study by Konken et al. (2003) illustrated that high temperatures were associated with an increased risk of hospital admissions for acute myocardial infarction and congestive heart failure but also with a decreased admission rate due to coronary atherosclerosis and pulmonary heart disease. Thus, combining all CVDs may have attributed to the decreased sensitivity in the relationship between temperature and CVD in this study. Third, this study failed to examine the modification effects of individual-level socioeconomic factors such as information on adaptive measures (e.g., air conditioning), occupation, or how much time people spent outdoors, which may reflect the actual exposure to high ambient temperatures. However, these factors may not change in a short period of time, as daily time series were used to examine temperature–health outcomes in this study. Finally, we did not control the effects of wind and air pressure (due to the lack of data) in the association between high temperature and mortality and hospitalization. Future studies should consider other weather factors (i.e., wind, air pressure) in examining the association between high temperature and health outcomes.

## 5. Conclusions

This study provided evidence revealing that while high temperatures significantly increase the risks of both mortality and hospitalizations for respiratory diseases, heat waves result in a significantly elevated risk of mortality among residents in the most populous city in Vietnam. In the comparison of effect magnitude, the heat-morbidity risk was less sensitive and certain than the heat-mortality risk. Future studies with larger-scale hospitalization data should be implemented to further examine the utility and sensitivity of admission data in terms of heat-health risk; it is also warranted to consider advanced methods that better model the uncertainty and overdispersion nature of time series data in further studies. HCMC is highly vulnerable to climate change; extreme temperature conditions, under interaction with other factors such as air pollution and socio-demographic factors (e.g., high population density), have been anticipated to more frequently occur in this city. Therefore, our findings have important implications for the projected impact on residents. Public health adaptation and prevention programs such as those aimed at developing early warnings and response plans, improving the capacity of healthcare systems to adapt to climate change-related extreme weather events, educating community members to minimize the effects of high temperatures, and establishing temperature shelters in residential hot spots should be implemented to protect residents from this additional burden of adverse health effects.

## Figures and Tables

**Table 1:** Summary statistics of daily weather conditions, daily mortality, and daily hospitalization in Ho Chi Minh City, Vietnam, 2010–2013.

Variables	Mean	Standard Deviation	Minimum	Percentile			Maximum
				25%	50%	75%	
Maximum temperature (°C)	33.8	1.8	24.5	32.7	34	35	39
Average temperature (°C)	28.4	1.3	23.0	27.5	28.4	29.4	32.1
Minimum temperature (°C)	25.4	1.4	20.0	24.5	25.4	26.3	29.8
Average relative humidity (%)	74.1	7.2	52	70	74	79	94
**Mortality data ^#^**							
*All-cause*	70	11.5	26	62	70	77	111
*Cardiovascular disease*	15	4.3	3	12	15	18	34
*Respiratory disease*	6.0	2.6	0	4	6	8	16
*Male*	38	7.3	13	33	38	43	64
*Female*	32	7.1	10	27	31	36	59
*0–14 years old*	1	1.0	0	0	1	2	5
*15–64 years old*	28	5.8	6	24	28	32	46
*≥65 years old*	41	8.6	14	25	40	46	71
**Hospitalization data ^#^**							
*All-cause*	222	52.3	83	175.8	224	258	456
*Cardiovascular disease*	42	9.3	18	35	42	48	76
*Respiratory disease*	25	7.1	7	20	24	30	49
*Male*	107	25.2	36	87	105	123	239
*Female*	115	29.4	26	89	116	136	220
*0–14 years old*	11	3.7	1	8	10	13	24
*15–64 years old*	56	15.1	19	44	55	66	112
*≥65 years old*	155	38.3	52	123	155	182	327

^#^ the unit of mortality and hospitalization is the number of cases per day.

**Table 2:** Intensity and duration of heat waves in Ho Chi Minh City, 2010–2013.

Heat Wave Definition (Threshold, Duration)	Start–End Date	Duration (Days)	Intensity (°C) Median (Range)
97th percentile, 2 days	24/4–26/4/2010	2	31.2 (31.07–31.25)
	5/5–21/5/2010	16	31.75 (30.95–32.1)
	23/5–27/5/2010	4	31.125 (30.925–32.125)
	30/5–2/6/2010	3	31.23 (31.025–31.3)
	31/3–6/4/2013	6	31.4 (30.9–32)

## Supplementary Materials

The following are available online at http://www.mdpi.com/1660-4601/16/3/432/s1: Figure S1: Alternative models for temperature–mortality relationship; Figure S2: Alternative models for temperature–hospitalization relationship; Figure S3: alternative models with different combination number of knots for temperature and lag days; Figure S4: The sensitivity results of the different heat wave definitions for mortality; Figure S5: The sensitivity results of the different heat wave definitions for hospitalization.
