# Supplementary material for: Effects of Extreme Temperatures on Mortality and Hospitalization in Ho Chi Minh City, Vietnam
Source: Int J Environ Res Public Health. 2019 Feb 2;16(3):432. doi: 10.3390/ijerph16030432 (PMC6388260; doi:10.3390/ijerph16030432)
Supplement: Supplementary file 1 [file ijerph-16-00432-s001.pdf]

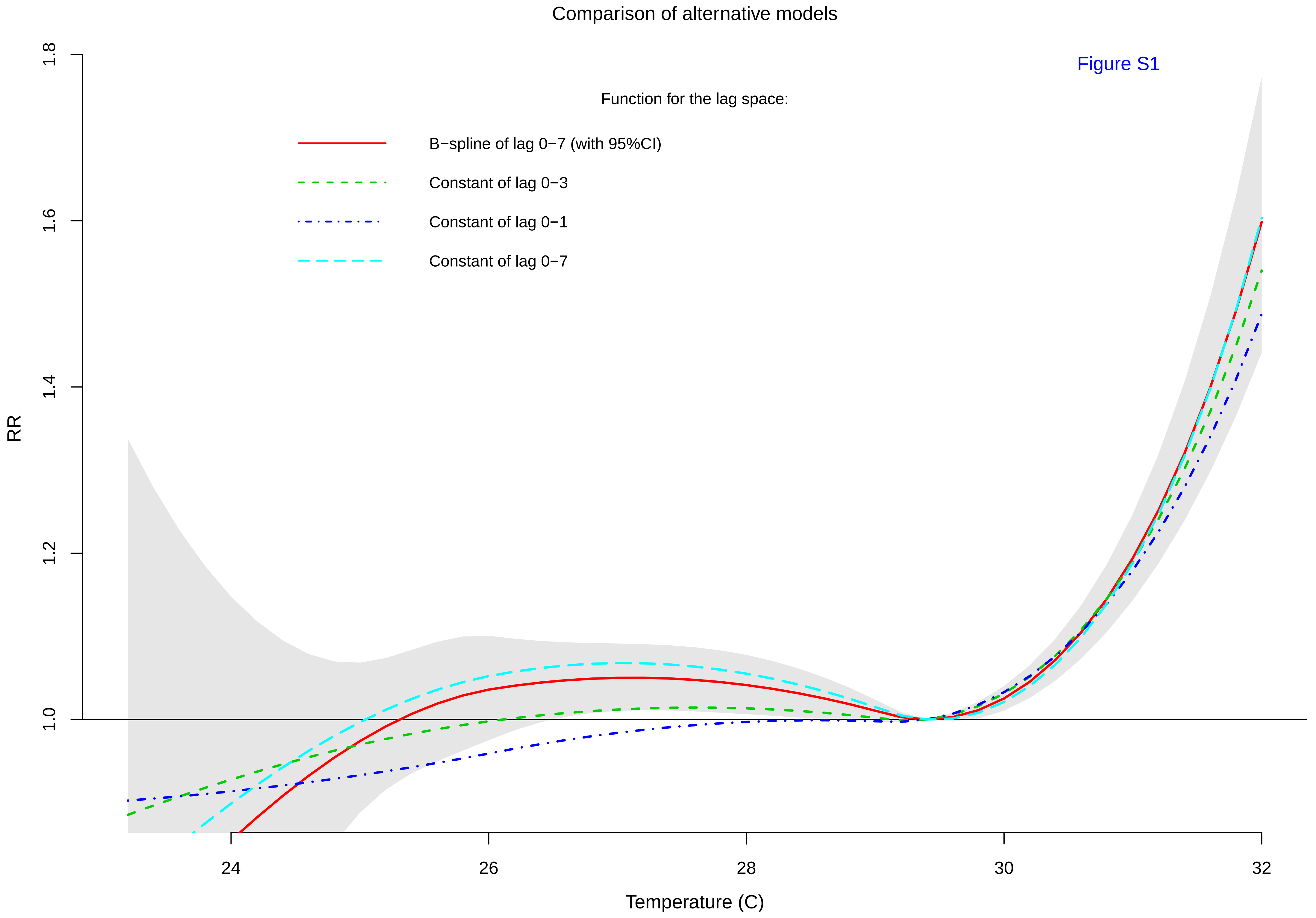
Comparison of alternative models
Figure S1
Function for the lag space:
B−spline of lag 0−7 (with 95%CI)
Constant of lag 0−3
Constant of lag 0−1
Constant of lag 0−7
RR
1.0
1.2
1.4
1.6
1.8
24
26
28
30
32
Temperature (C)

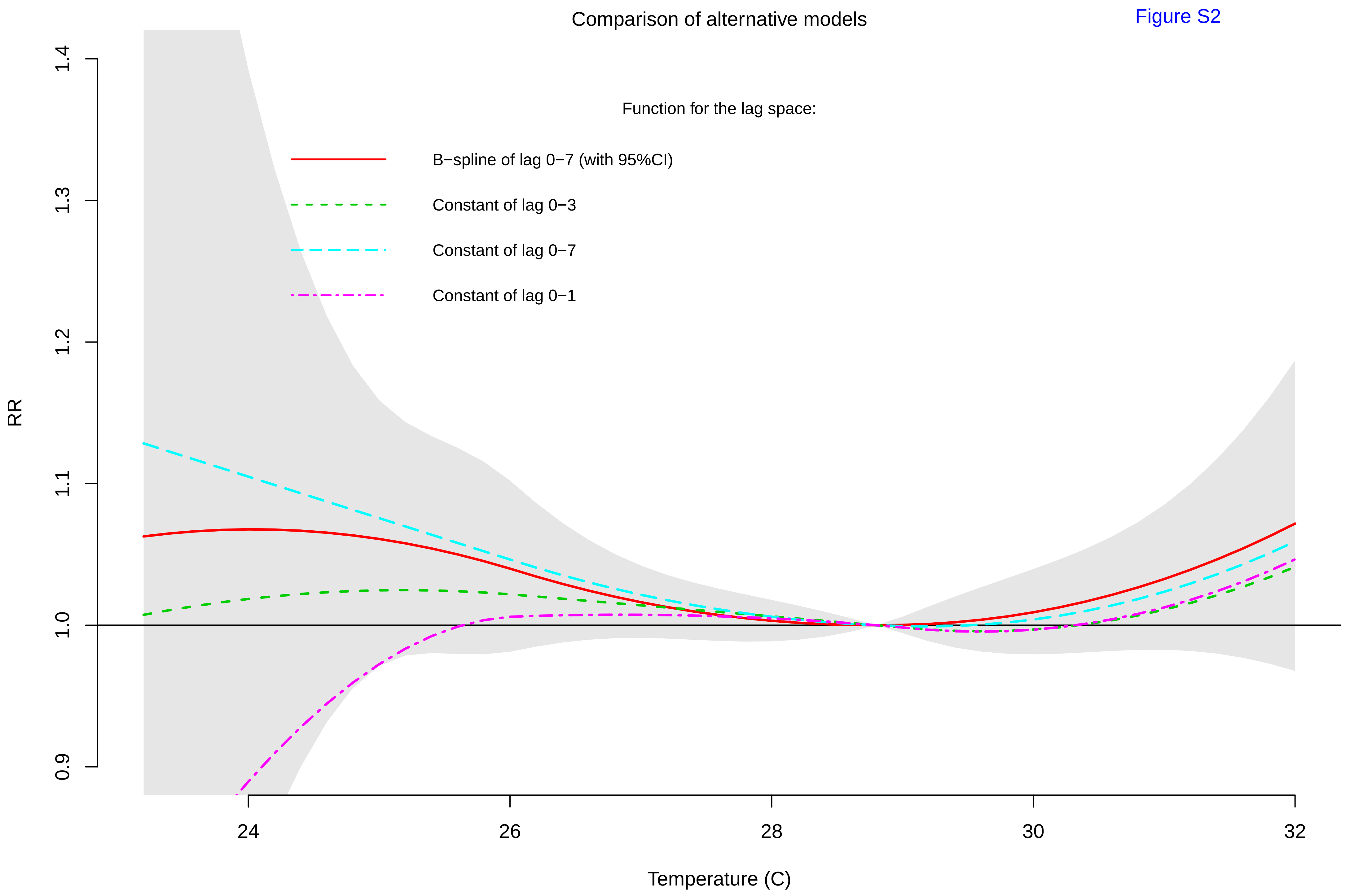

Comparison of alternative models
Figure S2
Function for the lag space:
B−spline of lag 0−7 (with 95%CI)
Constant of lag 0−3
Constant of lag 0−7
Constant of lag 0−1
RR
1.4
1.3
1.2
1.1
1.0
0.9
24
26
28
30
32
Temperature (C)

| | lag.1 | lag.2 | lag.3 | lag.4 |
|---|---|---|---|---|
| temp.1 | 11011.45 | 11000.29 | 11004.95 | 11008.12 |
| temp.2 | 11021.66 | 11011.27 | 11017.72 | 11020.19 |
| temp.3 | 11001.38 | 10990.42 | 10991.97 | 10994.48 |
| temp.4 | 11006.96 | 10991.94 | 10999.49 | 11007.52 |

**Figure S3a. The Q-AIC values of different combination of number of knots for temperature and lag days**

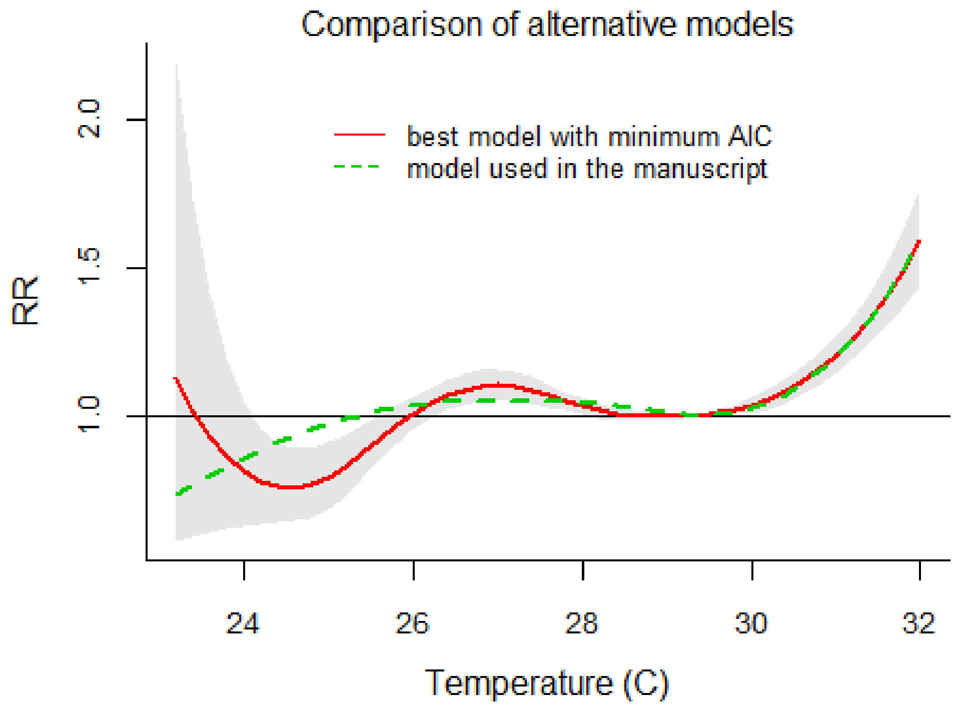


**Figure S3b. The comparison overall temperature-mortality association between the best model and the model used in the manuscript.**

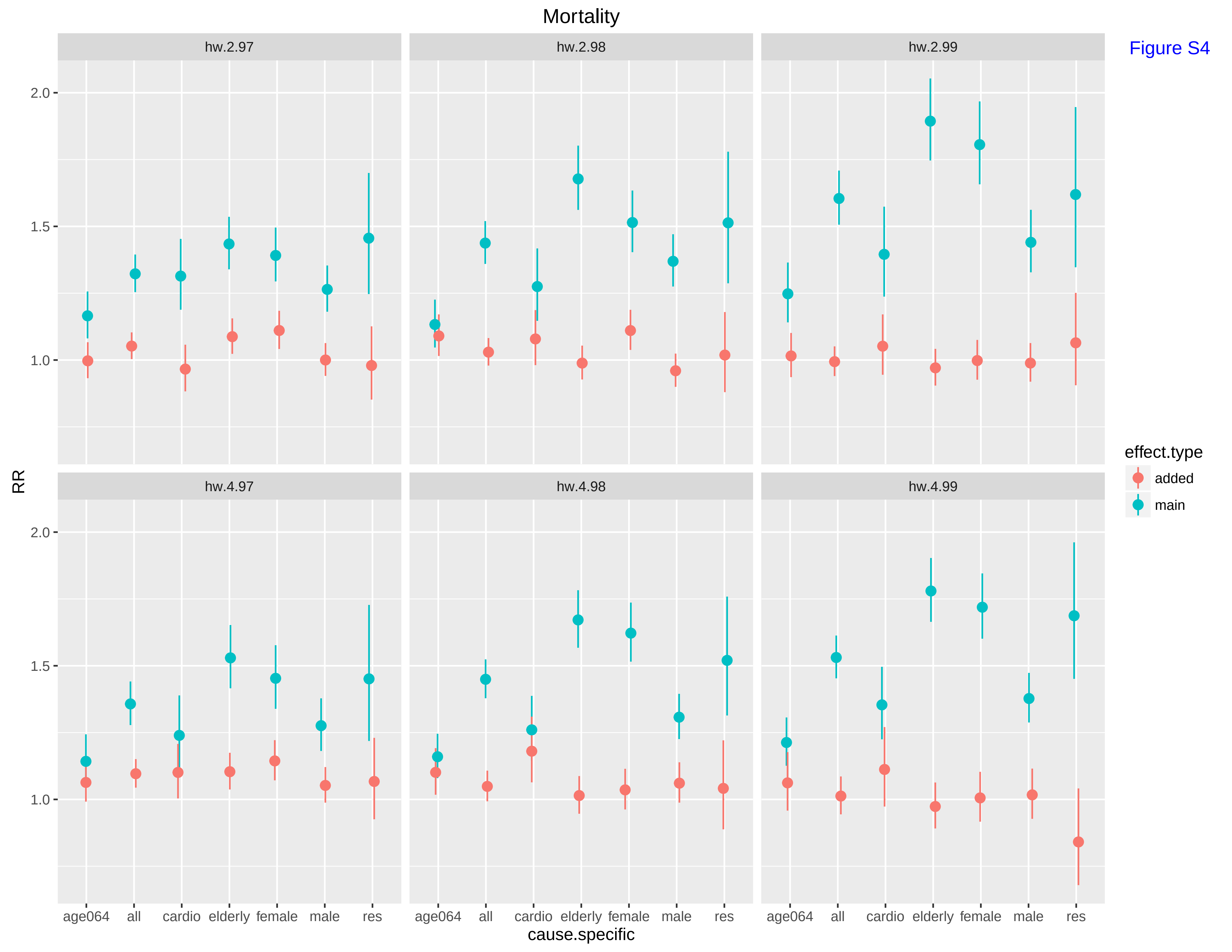
Mortality
Figure S4
hw.2.97
hw.2.98
hw.2.99
hw.4.97
hw.4.98
hw.4.99
2.0
1.5
1.0
RR
age064
all
cardio
elderly
female
male
res
cause.specific
effect.type
added
main

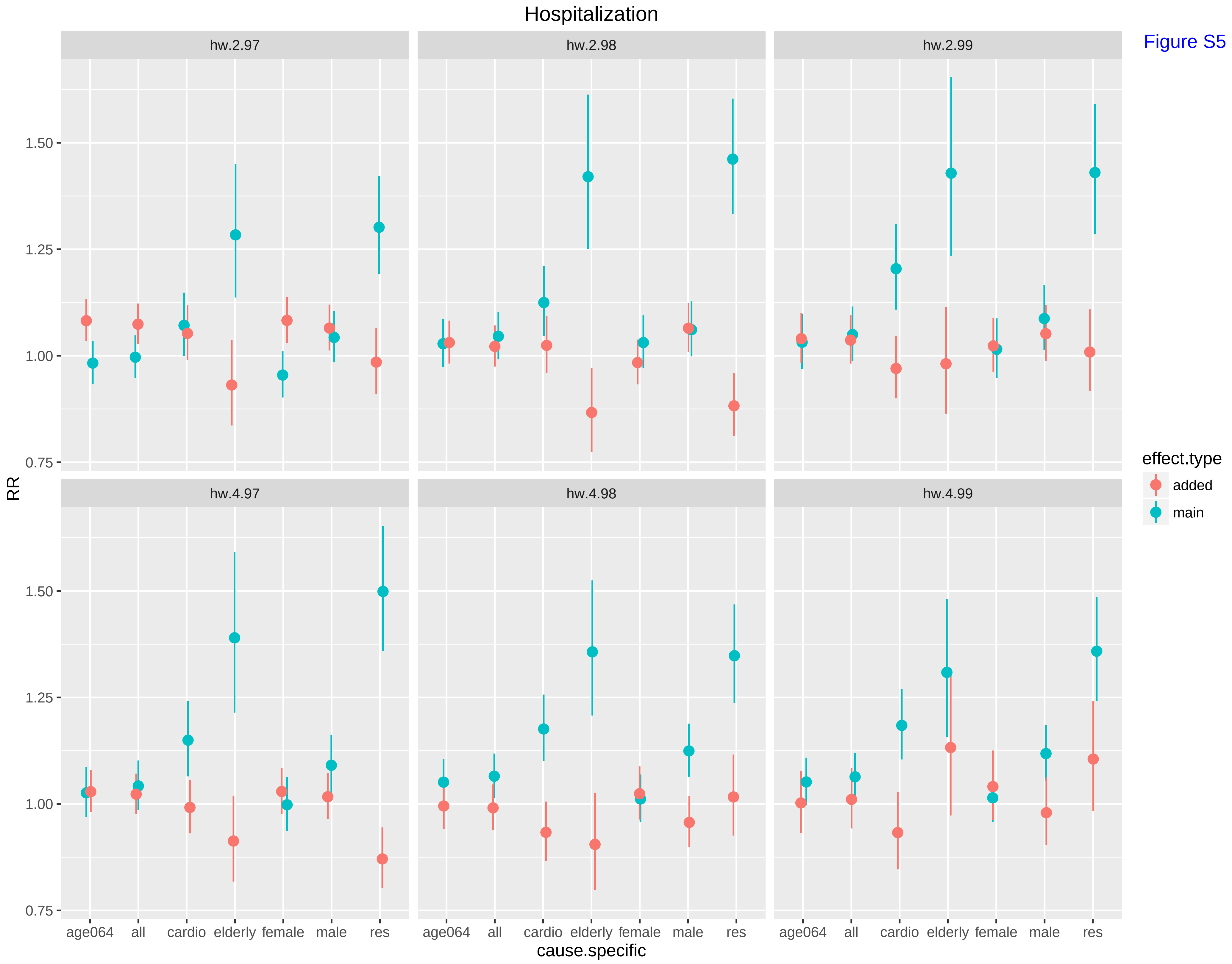

Hospitalization
Figure S5
hw.2.97
hw.2.98
hw.2.99
hw.4.97
hw.4.98
hw.4.99
RR
1.50
1.25
1.00
0.75
age064
all
cardio
elderly
female
male
res
cause.specific
effect.type
added
main
